# Association Between Lifetime Criminal Justice Involvement and Substance Use Disorders in U.S. Adults with Diabetes

**DOI:** 10.1089/heq.2021.0051

**Published:** 2022-09-07

**Authors:** Laura C. Hawks, Rebekah J. Walker, Leonard E. Egede

**Affiliations:** ^1^ Division of General Internal Medicine, Medical College of Wisconsin, Milwaukee, Wisconsin, USA.; ^2^ Center for Advancing Population Science, Medical College of Wisconsin, Milwaukee, Wisconsin, USA.

**Keywords:** diabetes, criminal justice involvement, mental health, Depression, Health Services Research

## Abstract

**Background:**

Criminal justice involvement (CJI) is a social risk in adults with both diabetes and substance use, however, the relationship between CJI, diabetes, and substance use disorders is not well studied.

**Methods:**

Data from a nationally representative sample of U.S. adults with diabetes from the National Survey of Drug Use and Health (2015–2018) were used to establish the prevalence of the following substance use disorders: alcohol, opioid, cannabis, cocaine, and methamphetamine, or a composite variable for any substance use disorder. Multiple logistic regression was used to test the association between CJI and each substance use disorder in adults with diabetes controlling for relevant covariates.

**Results:**

Of 11,594 respondents representing 25,834,422 U.S. adults with diabetes, 17.1% reported prior CJI. Prevalence of substance use disorders was significantly higher in individuals with CJI compared to those without CJI (alcohol: 8.3 vs. 2.2; opioid: 2.1 vs. 0.4; cannabis: 1.4 vs. 0.2; cocaine: 1.2 vs. 0.1; methamphetamine: 1.2 vs. 0.1; any substance: 11.86 vs. 2.78; *p*<0.001 for all). In fully adjusted models, odds of substance use disorders were significantly higher in individuals with CJI (alcohol: odds ratio [OR] 2.76, 95% confidence interval [CI]: 2.01–3.82; opioid: OR 5.08, 95% CI: 2.25–11.47; cannabis: OR 5.05, 95% CI: 2.60–9.81; cocaine: OR 23.62, 95% CI: 5.59–99.82; methamphetamine: OR 40.66, 95% CI: 13.23–124.95; any substance: OR 7.19, 95% CI: 4.47–11.56).

**Conclusion:**

In adults with diabetes, prevalence of substance use disorder is high among those with CJI. Interventions that target substance use disorders are needed in this population.

## Introduction

Diabetes mellitus is a common chronic disease in the United States, diagnosed in over 30 million U.S. adults, and is the seventh leading cause of death in the United States.^1^ Without optimal glycemic control, patients with diabetes are at risk of significant cardiovascular complications, including heart disease, end-stage renal disease, and lower extremity amputation.^2^ Comorbid substance use disorder, including alcohol or other drug use, is a major risk factor for poor glycemic control, diabetes complications, and mortality among individuals with diabetes. For example, having an alcohol use disorder has been associated with a 27% increased risk for diabetic neuropathy, 62% increase risk for myocardial infarction and a 35% increased risk of mortality among those with diabetes.^3^

There are several mechanisms by which substance use disorders may undermine diabetes management. One study examining Medicare and Medicaid claims data found that patients with substance use disorder received worse quality of diabetes care.^4^ In addition, self-management, which is care conducted by the patient in between clinic visits, is an important component to achieving glycemic control and substance use disorders likely undermine self-management behavior.^5^ Finally, complications of substance use disorder and diabetes may overlap, such as neuropathy for alcohol use disorder or cardiovascular disease for cocaine use disorder.^6^ Better understanding opportunities for clinicians to identify and treat comorbid substance use disorders will be important for improving glycemic control and long-term diabetes outcomes.

Criminal justice involvement (CJI) is a social risk, which is experienced by many patients with both diabetes and substance use disorder.^7,8^ CJI is associated with worse outcomes across the health spectrum, from infectious disease to chronic illness to behavioral health.^9–11^ Those with CJI are disproportionately members of minority groups and are more likely to have lower socioeconomic status, similar to those most likely to have poor health outcomes due to diabetes and substance use disorder.^12,13^ In recent years, additional research has been conducted examining the role of health care systems in improving outcomes for those with justice involvement.^14,15^ However, little is known about the relationship between CJI, diabetes, and substance use disorders.

To bridge this gap in knowledge, we used a nationally representative sample from the National Survey of Drug Use and Health (NSDUH) to establish the prevalence of substance use disorder among U.S. adults with diabetes and examine the association between CJI and the following substance use disorders in adults with diabetes: alcohol, opioid, cannabis, cocaine, and methamphetamine use disorder. We hypothesized that rates of these substance use disorders would be significantly higher in those with CJI and that prior CJI would be significantly associated with substance use disorders after adjustment for a robust number of sociodemographic and clinical confounders.

## Methods

Data from the NSDUH between the years 2015–2018 were used to create a nationally representative, cross-sectional sample of noninstitutionalized U.S. adults. The NSDUH is administered by the Substance Abuse and Mental Health Services Administration (SAMHSA). One role of the survey is to annually establish the prevalence of substance use and mental health conditions among adults and adolescents. The NSDUH also collects associated information, including self-reported chronic disease and CJI. Due to the sensitive nature of the questions, a computerized service is used to anonymously collect the majority of response. The survey response rate was between 66% and 69% during included years.^16^ The Medical College of Wisconsin's Institutional Review Board deemed this study exempt from review because the data are deidentified and publicly available.

### Study population (diabetes)

We considered all adults who reported a diagnosis of diabetes to be included in our study population. To identify adults with diabetes, we considered an affirmative response to the question, “Have you ever been told by a doctor or other healthcare professional that you have diabetes or sugar diabetes?”

### Main exposure (criminal justice involvement)

To identify participants by our main exposure variable, lifetime involvement in the criminal justice system, we considered responses to the question: “Not counting minor traffic violations, have you ever been arrested and booked for breaking the law? Being ‘booked’ means that you were taken into custody and processed by the police or by someone connected with the courts, even if you were then released.” Those who replied “yes” were considered to have prior CJI. We excluded missing responses from the study population.

### Outcomes (substance use disorders)

We identified six variables capturing past year substance use disorder. The NSDUH identifies respondents with substance use disorder using a questionnaire within the survey similar to the Diagnostic and Statistical Manual of Mental Disorders, 4th Edition (DSM-IV) to diagnostically predict substance use disorder.^17^ Using these DSM-IV criteria, the NSDUH model generates a diagnostic variable for abuse or dependence (hereafter described as “use disorder,” consistent with current DSM terminology) for the following substances: alcohol, opioids (including heroin and/or prescription opioids), marijuana (cannabis), cocaine, and methamphetamine.^18^

We additionally included a composite variable for any substance use disorder, which includes any of the following: alcohol, cocaine, heroin, prescription opioid, marijuana, hallucinogen, inhalant, methamphetamine, sedative, tranquilizer, stimulant, or psychotherapeutic drug. Each substance use disorder and the composite (any substance use disorder) were treated as separate outcomes.

### Covariates

To account for potential sociodemographic and clinical confounders, we included the a number of covariates in our multivariate regression models. Demographic covariates included age category (18–25, 26–34, 35–49, 50+); race/ethnicity (white [non-Hispanic], black [non-Hispanic), Hispanic, other [non-Hispanic Native American/Alaskan native, non-Hispanic native Hawaiian or Pacific Islander; non-Hispanic Asian; or more than one race]); gender (male, female).

Socioeconomic variables included poverty level (<100% federal poverty level as determined by the Census Bureau, 100–200% poverty level, >200% poverty level), educational status (less than high school, high school diploma or equivalent, some college, or college degree); marital status (married, widowed/divorced/separated, or never married); current employment status (full time, part time, unemployed, or other [disabled; keeping house full-time; in school/training; retired; or some other reason]); and insurance coverage (Medicare, Medicaid, VA healthcare, private, or none).

Our comorbidity variables included all self-reported physical medical conditions included in the survey: high blood pressure, heart condition, kidney disease, asthma, chronic obstructive pulmonary disease (COPD), cirrhosis, hepatitis B/C, HIV/AIDS, and cancer. Presence of each medical comorbidity was treated as a separate variable.

### Statistical analyses

First, we summarized the data using frequencies and conducted Pearson chi-square statistical tests to compare the sociodemographic characteristics and health profiles of those with or without lifetime CJI. We then estimated the prevalence of the sample population who met criteria for the included substance use disorders and compared prevalence between those with and without prior CJI.

Third, we calculated unadjusted odds ratios (ORs) using separate logistic regression models for each substance use outcome with CJI status serving as the primary independent variable. Finally, to investigate the independent association between CJI and substance use outcomes, we used multiple logistic regression again treating each substance use disorder as a separate outcome, CJI as the primary independent variable and adjusting for age, sex, race, poverty level, education, marital status, employment, health insurance coverage type, and comorbid medical conditions.

All analyses were performed using Stata version 16 (Stata Corp LP, College Station, TX, USA) and complex survey design was accounted for using weights provided by SAMHSA. Two-sided *p*-values of <0.05 were considered statistically significant.

## Results

Our sample included 11,594 respondents, which after weighting represented 25,834,422 adults with diabetes. Lifetime CJI in our sample was 17.1% (95% confidence interval [CI], 16.2–18.0). Table 1 reports the demographic characteristics by CJI status. Those with prior CJI were less likely to be aged 50+ than those with no prior CJI (69.5% vs. 78.3%, *p*<0.001), more likely to be male (72.2% vs. 42.8%, *p*<0.002) and more likely to be black (18.6% vs. 14.8%, *p*=0.02). They were also more likely report measures of low socioeconomic status, including an income of <200% federal poverty level (FPL) (47.3% vs. 39.8%, *p*<0.001) and an educational status of HS diploma equivalent or less (54.3% vs. 47.0%, *p*<0.001).

**Table 1.** Sample Demographic Characteristics of U.S. Adults with Diabetes by lifetime Criminal Justice Involvement, 2015–2018

	Overall (*n*=11,594)	Prior CJI (*n*=9384), %	No Prior CJI (*n*=2178),%	*p*
Age				<0.01
18–25	2.0	1.7	2.1	
26–34	4.3	5.2	4.1	
35–49	16.9	23.5	15.5	
50+	76.8	69.5	78.3	
Sex				<0.01
Male	47.9	72.2	42.8	
Female	52.1	27.8	57.2	
Race				0.02
White NH	60.5	59.9	60.7	
Black NH	15.4	18.6	14.8	
Hispanic	16.4	14.9	16.7	
Other	7.7	6.7	7.9	
Poverty level				<0.01
<100%	15.2	20.0	14.2	
100–200%	25.1	27.3	24.7	
>200%	59.7	52.7	61.1	
Education				<0.01
<High school	18.9	20.9	18.5	
HS diploma	29.4	33.4	28.5	
Some college	29.6	31.4	29.4	
College degree	22.1	14.3	23.7	
Marital status				<0.01
Married	57.5	48.8	59.3	
Divorced/widowed	29.4	32.3	28.8	
Never married	13.1	18.9	11.9	
Employment				<0.01
Full time	32.9	34.2	31.5	
Part time	9.9	8.4	10.2	
Unemployed	3.0	4.4	2.7	
Other^*^	55.2	53.0	55.7	
Health insurance				
Private	60.5	49.1	62.9	<0.01
Medicaid	19.7	27.5	18.1	<0.01
Medicare	46.8	39.3	48.4	<0.01
VA Health	9.0	11.7	8.4	<0.01
Uninsured	5.0	7.3	4.6	<0.01
Comorbidities				
Heart condition	23.8	26.1	23.4	0.09
Hypertension	45.3	42.5	45.8	0.02
Kidney disease	6.6	6.3	6.7	0.53
COPD	10.2	12.5	9.8	0.01
Asthma	11.3	11.2	11.3	0.94
Cirrhosis	1.2	1.8	1.1	0.08
Hepatitis B/C	2.2	4.8	1.7	<0.01
HIV/AIDS	0.2	0.5	0.2	0.06
Cancer	10.7	9.5	10.9	0.18

^*^ Other employment status includes disabled, keeping house full-time; in school/training; retired; or some other reason.

AMI, any mental illness; CJI, criminal justice involvement; COPD, chronic obstructive pulmonary disease; NSDUH, National Survey of Drug Use and Health; SUD, substance use disorder.

While those with prior CJI were less likely to have private insurance coverage or Medicare, they were more likely to have Medicaid, VA health, or be uninsured. In both groups there was a high prevalence of reported comorbidities, especially those related to diabetes, including a heart condition (26.1% vs. 23.4%, *p*=0.09), hypertension (42.5% vs. 45.8%, *p*=0.02), and kidney disease (6.3% vs. 6.7%, *p*=0.53).

Table 2 reports the prevalence of substance use disorders among U.S. adults with diabetes. Overall prevalence of substance use disorders in the study population was as follows: alcohol: 3.3%; opioid: 0.7%; cannabis: 0.4%; cocaine: 0.4%; methamphetamine: 0.2%, and any substance: 4.3%. Those with prior CJI were more likely to have each of the substance use disorders compared those never incarcerated. The difference in prevalence between those with prior CJI and those without was as follows: alcohol: 8.3 versus 2.2%; opioid: 2.1 versus 0.4; cannabis: 1.4 versus 0.2; cocaine: 1.2 versus 0.1; methamphetamine: 1.2 versus 0.1, *p*<0.001; and any substance use: 11.9 versus 2.8 (*p*<0.001 for all comparisons).

**Table 2.** Prevalence of Substance Use Outcomes Among Adults with Diabetes by Lifetime Criminal Justice Involvement, 2015–2018

	Overall (*N*=11,594), %	Prior CJI (*N*=9384), %	No prior CJI (*N*=2178), %	*p* ^a^
Substance use outcome				
Alcohol use disorder	3.3	8.3	2.2	<0.001
Opioid use disorder	0.7	2.1	0.4	<0.001
Cannabis use disorder	0.4	1.4	0.2	<0.001
Cocaine use disorder	0.3	1.2	0.1	<0.001
Methamphetamine use disorder	0.2	1.2	0.1	<0.001
Any substance use disorder	4.3	11.9	2.8	<0.001

^a^ *p*-value: comparing those with prior CJI to those with no prior CJI.

Table 3 presents the unadjusted and adjusted ORs of prior CJI for each substance use disorder. In our unadjusted logistic regression models, prior CJI was strongly associated with all substance use disorders in this sample of adults with diabetes. This association persisted after controlling for all sociodemographic and clinical comorbidities, including an adjusted ORs of 2.76 for alcohol use disorder (95% CI: 2.01–3.82), 5.08 for opioid use disorder (95% CI: 2.25–11.47), 5.05 for cannabis use disorder (95% CI: 2.60–9.81), 23.62 for cocaine use disorder (95% CI: 5.59–99.82), 40.66 for methamphetamine use disorder (95% CI: 13.23–124.95), and 3.55 for any substance use disorder (95% CI: 2.79–4.51).

**Table 3.** Association Between Substance Use Outcomes and Lifetime Criminal Justice Involvement Status Among U.S. Adults with Diabetes, 2015–2018

Substance use outcome	OR (95% CI)	Adjusted OR^a^ (95% CI)
Alcohol use disorder	3.95 (3.09–5.06)	2.76 (2.01–3.82)
Opioid use disorder	5.58 (3.11–9.99)	5.08 (2.25–11.47)
Cannabis use disorder	6.12 (3.70–10.43)	5.05 (2.60–9.81)
Cocaine use disorder	21.02 (7.43–59.43)	23.62 (5.59–99.82)
Methamphetamine use disorder	26.25 (8.44–81.62)	40.66 (13.23–124.95)
Any substance use disorder	4.70 (3.90–5.68)	3.55 (2.79–4.51)

^a^ Adjusted for age, sex, race, poverty level, education, marital status, employment, health insurance coverage type, and comorbid medical conditions.

CI, confidence interval.

## Discussion

This nationally representative sample of U.S. adults with diabetes, 500 respondents (weighted to represent about 1 million adults) had a comorbid substance use disorder. Moreover, the presence of both conditions is highly concentrated among those with prior CJI. Adults with diabetes and prior CJI were disproportionately members of minority groups and of lower socioeconomic status and were more likely to have Medicaid or be uninsured. Finally, our multivariate regression analyses found that prior CJI in adults with diabetes is highly associated with multiple forms of substance use disorder, findings that persist after controlling for sociodemographic and clinical confounders.

Our findings contextualize the small amount of existing literature on comorbid diabetes and substance use disorder. Prevalence studies have generally found that substance use is lower among those with diabetes compared with the general population, but that it undermines treatment when present.^3^ Only one other nationally representative study has examined the rate of alcohol use disorder among patients with diabetes; it found a prevalence rate of 1% (compared to a 3.3% prevalence in our study).^19^ To our knowledge, this is the first study describing prevalence of opioid use disorder in U.S. adults with diabetes. That substance use prevalence is particularly concentrated among those with prior CJI is not surprising (CJI is known to be strongly associated with substance use^7^), but these findings suggest that those with diabetes and CJI experience substance use disorders at rates higher than the general population.

Moreover, these findings taken in sum with prior data showing the harmful effects of substance use on diabetes outcomes shed light into potential areas for effective intervention.

Compared to other substance use disorders, alcohol and opioid use disorder (the majority of cases in this study) have the best evidence base for effective treatment regimens; however, there is a paucity of literature examining interventions which effectively treat these substance use disorders along with comorbid diabetes.^20^ One study examined contingency management, the provision of tangible positive reinforcement for abstinence from substances, in alcohol use disorder and found that patients with diabetes were more likely to achieve abstinence but did not examine diabetes outcomes.^21^ To our knowledge, there are no studies examining the effect of evidenced-based pharmacotherapy for alcohol use disorder such as naltrexone, disulfiram, or acamprosate in patients with diabetes.^6^

One small study in Canadian First Nations people with diabetes and opioid use disorder treated with buprenorphine-naloxone, a partial opioid agonist used to treat opioid use disorder (OUD), found substantial improvement in glycosylated hemoglobin in the treatment group.^22^ However, this study is not generalizable and only included 66 participants. Preclinical data suggest that methadone, a full opioid agonist used to treat OUD, may decrease glucose tolerance.^6^ Therefore, buprenorphine may be the preferable treatment option in candidates for opioid agonist therapy with diabetes, although additional clinical trials are warranted.

Our study shows that considering CJI as a social risk factor may facilitate treatment delivery in this population. However, current substance use treatment approaches for those currently involved in the legal system rarely align with best practices; there is no evidence finding that contact with the criminal justice system leads to improved access to effective, evidenced based care. For example, a recent study examining survey of court-mandated addiction treatment programs found that less than 10% record meaningful health-related outcomes.^23^ Another study found that only 5% of adults in the system are offered the first-line treatment for opioid use disorder, opioid agonist therapy.^24^ Most importantly, evidence suggests that a treatment-based approach is likely safer and more cost-effective than criminalization for managing substance use disorder.^25^

Our findings taken in sum with what was previously known about the negative impact of substance use on diabetic control underscore an urgency in prioritizing treatment for substance use disorder for those with comorbid chronic disease such as diabetes within criminal justice settings.

Although no studies have specifically examined interventions for those with diabetes and addiction after exposure to the justice system, a basis of evidence suggests that a primary care-based model may hold promise. Interventions for justice-involved patients reentering into the community, such as the primary care-based Transitions Clinic Network,^26^ may improve outcomes for those with both medical and behavioral health conditions. Similar interdisciplinary models, not specifically for the justice-involved, have been shown to improve outcomes when treating patients with several comorbid conditions.^27^ Based on our results, development of these interventions should consider how to incorporate factors related to prior CJI, and how this influences diabetes self-care behaviors and outcomes.

We acknowledge several limitations of our study. First, in this cross-sectional sample of data, causal association cannot be established. Second, because we used self-reported data on diabetes status to create our sample, those with undiagnosed diabetes are not included in the sample, and given the lack of health care access for CJI populations may suggest that CJI was overrepresented in these missed cases.^28^ However, this bias would render our findings more conservative. Third, the CIs were wide for some estimates, due to the small sample size; therefore, these estimates should be interpreted with caution. Finally, the CJI measure is also self-reported and may be underreported; however, other studies have shown this to be a reliable method for attaining such information.^29^

In conclusion, over one million U.S. adults are living with both diabetes and substance use disorder, and coprevalence is concentrated among those with CJI. CJI was strongly and independently associated with substance use outcomes even after controlling for multiple sociodemographic and medical covariates. Leveraging resources to improve access to both SUD and diabetes treatment is needed to improve health outcomes in the justice-involved population.

## Abbreviations Used

CI: confidence interval
CJI: criminal justice involvement
DSM-IV: Diagnostic and Statistical Manual of Mental Disorders, 4th Edition
NSDUH: National Survey of Drug Use and Health
OR: odds ratio
OUD: opioid use disorder
SAMHSA: Substance Abuse and Mental Health Services Administration
